# Exploration of Microencapsulation of Arginine in Carnauba Wax (*Copernicia prunifera*) and Its Dietary Effect on the Quality of Beef

**DOI:** 10.3390/ani14131857

**Published:** 2024-06-23

**Authors:** German Contreras-Lopez, Ivan A. Garcia-Galicia, Luis Manuel Carrillo-Lopez, Agustin Corral-Luna, Lorenzo Buenabad-Carrasco, Mieke Titulaer, José A. Villarreal-Balderrama, Alma D. Alarcon-Rojo

**Affiliations:** 1 Facultad de Zootecnia y Ecología, Universidad Autónoma de Chihuahua, Perif, Francisco R, Almada km 1, Chihuahua, Chihuahua 31453, Mexico; jerman_219@hotmail.com (G.C.-L.); acorral@uach.mx (A.C.-L.); lbuenabad@uach.mx (L.B.-C.); mtitulaer@uach.mx (M.T.); jvillarreal@uach.mx (J.A.V.-B.); aalarcon@uach.mx (A.D.A.-R.); 2 C.E.I.E.G.T., Facultad de Medicina Veterinaria y Zootecnia, Universidad Nacional Autónoma de México, km. 5.5 Carr. Fed, Martínez de la Torre-Tlapacoyan, Tlapacoyan 93600, Mexico; 3 Consejo Nacional de Humanidades, Ciencia y Tecnología, Av. Insurgentes Sur 1582, Col. Crédito Constructor, Del. Benito Juárez, Ciudad de México 03940, Mexico

**Keywords:** beef quality, microencapsulation, arginine, dietary supplementation

## Abstract

**Simple Summary:**

Arginine is an important amino acid for cattle, as it can help improve their immune response and the amount of fat in their meat. However, when arginine is given to cattle through their food, bacteria in their stomach can use it up before it has a chance to benefit the animal. In a recent study, researchers looked into whether adding a special protective coating to the arginine, using carnauba wax, could change the color, tenderness, and fat content of beef in three different breeds of heifers. We found that adding this protected arginine to the cattle’s diet made the meat redder, more tender, and fattier after it was aged for 28 days. However, we also found that the breed of the cattle had a big impact on these qualities, with meat from Angus cattle being the most tender, even though it had slightly less fat compared to Hereford or Angus × Hereford crossbreeds. Hence, we concluded that both the protected arginine and the breed of the cattle can affect the quality of the beef.

**Abstract:**

The objective of this exploratory study was to assess if microencapsulated arginine influences the physicochemical quality of beef. The study included three genetic groups: Angus, Hereford, and Angus × Hereford crossbreed. Two encapsulation systems were used with carnauba wax, at ratios of 3:1 and 2:1, carnauba wax:core (arginine), respectively. A control treatment was also included with no arginine addition. Encapsulated arginine with a 3:1 ratio increased redness by 19.66 at 28 d aged beef compared to the control and 2:1 ratio with values of 18.55 and 16.77, respectively (*p* = 0.01). Encapsulated arginine at a 3:1 ratio showed the lowest meat shear force values with 24.32 N at 28 d of ageing (*p* < 0.001). The Angus breed also had a low value of 24.02 N (*p* < 0.001). Finally, the highest values of intramuscular fat were observed with the inclusion of arginine in a 3:1 ratio. The fat value reached 2.12% with a 3:1 ratio (*p* = 0.002), while in the Angus breed it was 1.59%. The addition of carnauba wax-encapsulated arginine can improve meat quality. It enhances red color, tenderness, and marbling in bovine meat.

## 1. Introduction

The global population is expected to grow in coming years, which will result in an increase of global food consumption. Specifically, meat consumption is estimated to rise by around 14% by 2030 [1]. The necessity to produce more meat in the present and the near future requires the search for strategies to make the use of ingredients in diets of animals producing the meat more efficient. In this regard, high quantities of metabolizable protein (MP) in the diets are needed to maximize lean tissue deposition in animals, which often surpasses the protein supply in the diet [2].

Including bypass amino acids in the diets of cattle improves productive performance [3]. This dietary strategy helps to limit the amino acid degradation in the rumen by ruminal microorganisms so they can instead be used for muscle deposition [4]. Efficient utilization of all resources in meat production is important. Livestock feed additives contribute to high environmental and economic costs, either by gas production or/and excretion of ammonia in feces to the environment. Certain ingredients as additives in the ruminant diets like amino acids can improve their performance, productivity, and product quality. Nevertheless, these types of additives must be protected from ruminal microorganisms to be beneficial to the animal. One way to protect these ingredients is through microencapsulation. For instance, arginine supplementation increases intramuscular fat (IMF) and fatty acid (FA) content deposition without affecting the proportions and ratios of the most important FAs, such as n-6:n-3 in pork [5,6]. In addition to increasing the marbling, dietary arginine has shown to decrease cooking and drip losses in pork [7]. L- arginine in the diet also enhances the expression of genes related to the growth of muscle fibers, and the accumulation of fat among muscle bundles [8]. Further, supplementation of dietary arginine at 1% in ruminants (sheep) has been reported to improve the productive performance and trigger the muscle protein deposition by expression of myogenin, myostatin, and muscle atrophy F-box, which directly impact on the increase in the loin eye area in lamb [9]. This could be counterproductive for IMF deposition because the change in metabolism reduces the lipogenesis through an effect of myostatin expression depletion. The impact of arginine on gene expression rather than an improved use of nutrients by the ruminal microflora has been confirmed in studies reporting no effect of 360 or 180 mg rumen protected L-arginine on acetate, propionate, or butyrate concentrations in the bovine rumen, despite an increased intestinal arginine flow and digestibility of the amino acid [10]. The improvement of gene expression by arginine in different species leads to higher meat quality. Nevertheless, the effects of rumen bypass arginine on meat quality remain unclear.

Microencapsulation involves embedding a substance in another material using physical or mechanical processes [11]. Environmental factors such as oxygen, water, pH, and interactions with other ingredients can impact the stability of active compounds [12]. Encapsulation processes are used in chemical, pharmaceutical, and food industries. They protect active compounds and achieve controlled release. They also reduce adhesion during storage and transportation and prevent changes in properties [13]. The encapsulation process in animal nutrition was introduced during the 1990s, with a wide exploration during the last 20 years [14]. Nonetheless, its applications seem very distant from the real potential compared to other industries such as the pharmaceutical industry. The actual uses of microencapsulation in animal nutrition aim to improve the delivery of helpful substances in the intestine, such as fatty acids, amino acids, antioxidants, and enzymes. Additionally, this can also include live microorganisms such as probiotics [15]. Yet, the use of microencapsulation in the animal industry remains vastly unexplored and it is necessary to expand the possibilities of the application that benefit the efficient production of food from animal origin in a more ecological approach.

Due to its hydrophobic characteristics, Carnauba wax (*Copernicia prunifera*) has shown to efficiently encapsulate amino acids and other nitrogen components such as urea cores against ruminal microbial degradation, as bypass nutrients. Carnauba wax has as advantages its high availability in the market, low cost, and it is nontoxic to animals. It is an encapsulant highly utilized in the medicine, dentistry, automobile, and food industries [16,17].

The objective of this exploratory study was to evaluate the effects of a low cost microencapsulation of arginine within carnauba wax at two different proportions (wax–arginine, 3:1 and 2:1) supplemented in bovine diets (Angus, Hereford, and Angus × Hereford) on the physicochemical quality of the meat, before and after retail simulation of 7 d.

## 2. Materials and Methods

### 2.1. Characteristics and Origin of the Animals and Samples

This study was carried out in the Biochemistry laboratory and feedlots of the School of Animal Science and Ecology of the Universidad Autonoma de Chihuahua (UACH). Fifteen heifers in total (Angus, Hereford, and Angus × Hereford crossbreed, 357 ± 24.6 kg initial weight) produced in the Teseachi experimental ranch from UACH were used in this experiment. The handling and housing, as well the experimental procedures with the animals complied with the institutional Bioethics code and Animal Welfare Guidelines, approved by the Institutional Bioethics and Animal Welfare Committee with authorized number P/302/2017. All animals were weighed at the start and end of the trial and daily weight gain was calculated to determine if the addition of microcapsules affected the productive performance.

### 2.2. Evaluated Treatments

The heifers were assigned randomly to one of the three treatments: T1, control without arginine; T2, microcapsules 3:1 wax–arginine ratio; and T3, microcapsules 2:1 wax–arginine ratio. Fifteen animals in total were evaluated considering five animals in each treatment. Every treatment had heifers from the three breeds. The animals were supplemented daily for 28 d prior to sacrifice with 50 g of microencapsulated arginine. The diet was formulated according to nutritional requirements by the Committee on Nutrient Requirements of Beef Cattle [18] and it was offered daily ad libitum to the animals. The composition is presented in Table 1.

The animals were slaughtered at a Federal Inspection slaughterhouse in Chihuahua city. The slaughter procedure followed Official Mexican regulations (NOM-033-SAG/ZOO-2014) [19]. The *Longissimus lumborum* muscle caudal to the 13th rib was dissected directly from the carcass to be used in the analysis of variables. The muscles (20 cm, aprox.) were vacuum packed and transported with a refrigerated vehicle (20 min at 4 °C) to the meat laboratory at UACH, twenty-four hours postmortem. Once received, the samples were sliced into 2.54 cm cross sections. The *Longissimus lumborum* sections were re-vacuum packed in a vacuum bag (70 µm thickness, polypropylene) and aged for 28 d (4 °C, 55% humidity) and physical–chemical measurements were carried out. After 28 d aged some samples were analyzed while others were packed in commercial polystyrene trays (21 cm × 18 cm) with commercial meat soaker pads and wrapped with commercial polyvinylchloride film permeable to oxygen (Food grade- 6 cm^3^ m mm m^−2^ d atm^−1^ at 23 °C) to simulate a test of shelf-life in retail display conditions, and left refrigerated at 4 °C, in darkness for 7 d. After the simulation of retail exposure, the physical–chemical measurements were carried out again. The variables evaluated were pH, water retention capacity (WHC), shear force, CIEL*a*b* space, and intramuscular fat content.

### 2.3. Microcapsules Production

The microcapsules production was performed at the Biochemistry laboratory of the School of Animal Science and Ecology of the UACH. The carnauba wax was purchased from “Ceras universales S.A.” a Mexican distribution center (https://cerasuniversales.com/ accessed on 12 April 2024). The L-arginine (CAS No. 1119-34-2) was obtained from ”Encapsuladoras de México, S.A. de C.V.” (https://encapsuladoras.com accessed on 12 April 2024), which conforms to the standards FCC11 and USP41.

The melt emulsion technique was performed to encapsulate arginine in carnauba wax. The wax was melted at 90 °C and the arginine was added with constant stirring at 500 rpm until complete homogenization. Span 80 ^®^ (Sorbitan Monooleate, Supelco ^®^, Bellefonte, PA, USA, 2.5% of the volume of water) was added into 200 mL of distilled water heated at the same temperature than wax. Water was added to the arginine dispersion with the wax and sped at 500 rpm for 3 min. The mix was allowed to cool to room temperature and crushed with a mortar.

### 2.4. Morphological Characterization

To evaluate and characterize the microencapsulated systems, micrographs were obtained by scanning electron microscopy at 100×, 500×, 1500×, and 5000× (SEM). SEM micrographs of the samples were obtained using a JSM-6390 SEM scanning electron microscope (Jeol, Tokyo, Japan). The size of the microcapsules of the different microencapsulated systems was determined with the Imagej program.

### 2.5. Physicochemical Evaluation

Evaluation of the physicochemical meat characteristics was performed after the ageing period (28 d post-mortem) and after the simulation of retail display (7 d).

pH. The pH was evaluated with a digital potentiometer for meat (Sentron, Model 1001, The Netherlands). The measurements were taken directly on the meat according to the method of Honikel [20]. The electrode was inserted perpendicular to the muscle to a depth of 2 cm, avoiding contact with the remaining fat and connective tissue. Three readings were taken in different areas of the sample and the average was calculated.

Water Holding Capacity (WHC). Exudate release was determined by the compression method proposed by Tsai and Ockerman [21], using 0.3 g of sample compressed 20 min. An analytical balance with a resolution of ±0.05 g, filter paper (#1 Whatman^®,^ Maidstone, UK), and 2.25 kg methacrylate plates were used. The results were expressed as a percentage of exudate released, according to the following expression: % exudate = (weight of the sample after compression) − (weight of the sample before compression)/weight of the sample before compression × 100. The determinations were performed by triplicate on each sample.

Shear Force. The samples were prepared for the shear stress according to the AMSA methodology [22]. The samples were cooked on electric griddles (George Foreman^®^, Beachwood, OH, USA) until reaching an internal temperature of 71 ± 0.1 °C, they were stored for 12 h at 4 °C and eight 10 mm diameter cylinders were obtained using a manual punch. The cylinders were obtained parallel to the longitudinal orientation of the muscle fibers. These were cut using a Warner–Bratzler blade (60 ° triangular opening) at a speed of 100 mm/min into 30 mm lengths. The peak force (expressed in kg-force) for the transverse cut in each cylinder was determined on a TA-XT plus texture analyzer (Stable Micro Systems Ltd., Godalming, UK).

Color (CIEL*a*b*). The color space was determined by the CIEL*a*b* parameters, where L* is luminosity, a* (+) is the red tendency and b* (+) expresses the yellow tendency. The measurements were obtained with a colorimeter (Konica Minolta CR400, Ramsey, NJ, USA. Illuminant C. D_65_. 2° observer angle of measurement. Aperture of 8 mm) according to the CIE (Commission Internationale Pour I`Eclarige) reference system [23] and the AMSA methodology [24]. For this, connective tissue and visible fat were removed from the muscle surface and the samples were exposed to air oxygen for 30 min to allow oxygenation of myoglobin (blooming). Three readings were taken for each sample in different areas and averages were obtained for the values of L*, a*, b*.

Total lipid extraction. The extraction was carried out with the Soxhlet method described by the Association of Analytical Chemists, 1995 [25].

### 2.6. Statistical Analysis

A completely randomized experimental design was used. The evaluated variables were analyzed with Analysis of Variance (ANOVA) using PROC GLM of the SAS System v.9.0 statistical package. Differences among treatments were evaluated using Tukey’s mean comparison test (*p* < 0.05). Values were reported as means ± standard deviation.

## 3. Results

### 3.1. Morphological Characterization

**Figure 1:**
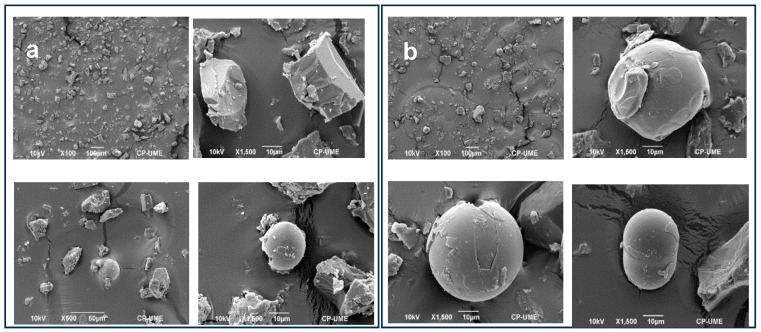
Micrographs of the microcapsules synthesized from the wax–arginine formulation. Formulation (**a**) corresponds to 2:1 formulation wax–arginine; formulation (**b**) corresponds to 3:1 formulation wax–arginine.

Figure 1 shows the morphology of the wax–arginine formulations. Both formulations presented two morphologies with crystals and microcapsules. The diameters of the microcapsules for the 2:1 and 3:1 ratio were 22.61 ± 0.81 and 48.73 ± 2.25 µm, respectively. The 3:1 wax–arginine formulation was observed to form more microcapsules compared to the 2:1 wax–arginine formulation. The crystals showed a smooth and flat surface, and the microcapsules had a regular spherical shape, although some of them had irregular shapes and smooth surfaces, while some others cracked. This last effect could be due to the crushing before the inclusion in the animal diet.

### 3.2. Weights and Gains

Final weights and weight gains during the trial are presented in the Table 2. No significant differences were observed and that was expected since the trial lasted a short period of 28 d. Nevertheless, the weights were taken to determine the amount of feed to offer to the animals and to calculate the weight gains.

### 3.3. pH

The pH of the beef was not significantly different (*p* > 0.05) among arginine treatments. The results for the pH in meat after ageing are shown in Table 3. In the case of the retail-exposed samples, again, no significant differences were found among treatments (Table 4, *p* > 0.05). Nonetheless, the pH of beef seems to increase after being exposed to simulated retail display permeable to oxygen, with values of 6.37, 6.25, and 6.31 for the control, 3:1, and 2:1 ratio, respectively. Additionally, differences in pH were observed between Angus and Angus × Hereford (6.54 and 6.57, respectively) compared to Hereford (5.81), after retail exposure (*p* < 0.001) (Table 4).

### 3.4. Water Holding Capacity (WHC)

No differences in WHC were found among treatments after the aging or oxygen permeable storage. The WHC values are presented in Table 3 for aged beef and in Table 4 for aged + retail display beef. Different WHC were found among genetic groups only in aged meat (Table 3); nonetheless, that difference was not persistent after the seven days of simulated permeable to oxygen storage (Table 4). The meat from Angus × Hereford had the highest value with 73.14% of WHC.

### 3.5. Intramuscular Fat (IMF)

Significant differences were observed among treatments and racial groups in intramuscular fat content (Table 3). The encapsulated arginine treatments had the highest values compared to the control. The 3:1 ratio had the highest value of intramuscular fat (2.12%). In addition, the Angus animals resulted with the highest values of IMF content (1.59%) compared to the other two racial groups.

### 3.6. Shear Force

The shear force decreased significantly (*p* < 0.05) in the samples aged with the inclusion of the encapsulated arginine treatments compared to the control (Table 3). The 3:1 ratio obtained the lowest shear stress value (24.32 N). However, no difference (*p* > 0.05) among the encapsulated arginine treatments was observed. Regarding the breed, the meat from Angus × Hereford crossbreeds had the highest values (*p* < 0.05) of shear force (30.4 N) compared to the other two breeds. In other words, the meat from the crossbreed was tougher in comparison with the other two groups. After the retail exposure, the samples continued being significantly different (*p* < 0.05). The meat tended to reduce the shear force after the exposure to oxygen during the seven days of storage (Table 3 and Table 4).

### 3.7. Color

No significant differences were detected in none of the color coordinates (L*, a*, or b*1), either after aging or aging + retail display (Table 5 and Table 6, respectively). Nevertheless, a tendency to loss of color can be observed after the beef was exposed to oxygen permeable wrapping (reduction in shelf-life).

## 4. Discussion

### 4.1. Morphology of Microcapsules

Smaller particles with an irregular and porous surface can accelerate the release process of microencapsulated components into the animal system [17]. This has been observed in encapsulated urea in carnauba wax at three proportions 1:2, 1:3, and 1:4. The morphology of the particles of the wax–arginine formulations does not directly affect the physicochemical characteristics of meat quality, but they can be affected by a more rapid release in the rumen and the arginine being used by microorganisms, instead of being absorbed in the intestine. Nevertheless, the rapid release in the rumen does not occur because carnauba wax does not undergo the hydrolysis and biohydrogenation processes of microorganisms [16].

### 4.2. Weights and Gains

L- arginine as a supplement in the feeding of poultry, pigs, sheep, and cattle has shown a potential capacity to improve the productive performance parameters through an increase in protein deposition in muscles, lipid metabolism change, and a raise of digestibility of forage in ruminants [4,7,9,26,27]. However, supplementation with L-arginine does not modify the feed conversion ratio in cattle [2]. This is confirmed by a recent review of arginine in nutrition and metabolism in ruminants, which particularly mentioned that there is a big opportunity for research on encapsulated arginine in ruminant feeding and nutrition [28]. Furthermore, the studies reporting benefits from supplementing arginine in the diet did it for longer periods (21 d in poultry and 60–170 d in other species), in comparison to the present study (28 d). Hence, we hypothesize that the effect of arginine supplementation on productive performance parameters is visible when the animals are exposed for longer times. In short times such as 28 d, the supplementation of arginine was only reflected on the composition of the muscle.

### 4.3. pH

Ageing of beef increases pH values according to Tuell et al. [29] who included rumen bypass arginine and found that aging the meat for 28 d increased the pH. In the present study, a trend towards higher pH values can be observed after the aged beef was subjected to retail display. In contrast, Cottrell et al. [30] reported a reduction in pH in muscle Semimembranosus of Border Leicester × Merino lambs at 3 h, but not at 24 h postmortem. This reduction in the pH was observed after the administration of 500 mg/kg bolus arginine via a jugular catheter, and 190 min before their slaughter. The reduction in the pH could be related to a decrease in lactic acid content as postmortem glycolytic activity slows down along with amine formation [31]. This may be due to an increased presence of the amino acid arginine in their injected animals. However, the dietetic administration of arginine for 28 d in the present study did not produce any change in the pH of beef.

The lowest pH value in the beef from Hereford animals in the present study may be due to a possible difference of glycolytic activity in the muscle fibers, derived from variations among breeds. Usually, muscles with high glycolytic activity tend to develop a lower pH after ageing. Differences of glycolytic activity in muscle among breeds are reported and they may impact the quality of the meat initially by slight modifications of pH [32,33].

The observed increase in the pH after retail display (oxygen exposure) may be derived from the oxidation of protein and lipids that can produce chemical components such as ammoni, amines, nitrosamines, etc. that can lead to a rise in pH [34,35].

### 4.4. WHC

Despite a slight trend towards a decrease in WHC in the beef from cattle supplemented with arginine (2:1), the results of WHC in this study are in contrast to those obtained by Ma et al. [5]. They reported an improvement in WHC in pork loins when supplemented with 1% arginine in the diet. Also, the results obtained by Tuell et al. [29] showed an improvement of WHC in beef loins aged for 28 d by supplementing 10 g/d of ruminal bypass arginine for 180 d to cattle. Additionally, Madeira et al. [36] also reported a reduction in cooking losses in *Longissimus lumborum* of Large White × Landrace female pigs. This reduction was observed after supplementing arginine to the diet of the pigs in comparison to animals without arginine supplementation as a control treatment. Cooking losses in meat are frequently related to a higher WHC.

As mentioned in the previous paragraph, genetic differences of breeds can produce differences in the proportions of muscle fiber types among the animals. This consequently produces differences of physicochemical properties such as pH or WHC among breeds. In this regard, high proportions of muscle fibers type I or slow oxidative fibers are related to a slow decrease in pH derived from an aerobic respiration after the slaughter of the animal, which results in a higher WHC in the muscle [33,37]. In other words, higher glycolytic activity in the muscle originating from differences between breeds may cause a decrease in pH [32,37].

### 4.5. IMF

The effect of arginine supplementation on intramuscular fat observed in the present study coincides with the results reported by Ma et al. [5]. They reported an increase in IMF in pork loins, after supplementing 1% of arginine in the diet of pigs, until reaching a live weight of 100 kg. Furthermore, Teixeira et al. [4] reported an increase in marbling in the loins of Angus × Simmental bovines after their supplementation of 10 g/d of ruminal overflow arginine during 180 d. Additionally, they reported an increase in the proportion of choice quality grade in meat from the same animals supplemented with the arginine, in comparison to those not supplemented.

Guo et al. [6] also found an increase in IMF content in Duroc x Large White x Landrace pigs when supplemented arginine at 1% + glutamic acid, compared to a control corn/soybean meal-based diet. The increase in IMF by the addition of the amino acid in the diet can be attributed to the fact that arginine upregulates the expression of lipogenic genes such as fatty acid synthase (FAS) and peroxisome proliferator-activated receptor γ (PPAR γ) [38]. In this regard, Choi et al. [8] confirmed the increase in fatty acid synthase (FAS) in Angus steers after 14 d of abomasal infusion of arginine. This effect indicates an increased lipogenic capacity, which consequently benefits the deposits of IMF. This effect has been also confirmed in uricotelic species which cannot synthesize arginine. Wu et al. [39] reported an increase in intramuscular fat content in duck breast muscle when supplementing dietary arginine at 10 g/kg arginine. Meanwhile, arginine supplementation reduced subcutaneous and abdominal fat in the carcass. The authors attribute this re-configuration of fat in the carcass to a reduction in hepatic lipogenic enzyme activity caused by arginine.

In contrast, Tous et al. [40] reported a reduction in IMF in (Landrace × Duroc) × Pietrain pigs supplemented with arginine with a reduced protein diet. The argument of the authors was based on the hypothesis that the effect of arginine on IMF depends on the dietary level of lysine and leucine. This was also suggested by Madeira et al. who point out that to increase the IMF the level of lysine in the diet was more critical than the level of protein [41]. Hence, the effect of arginine in promoting the expression of adipogenic genes and lipid deposition is not definitive and depends on the way it interacts with other amino acids such as lysine and leucine.

### 4.6. Shear Force

Regarding the shear force or the tenderness of the beef in the present study, comparable results were observed by Ma et al. [5] and Tuell et al. [29]. In both studies the authors reported that the inclusion of arginine in the diet of cattle and pigs reduced the cutting effort of meat samples compared to the control. Arginine supplementation in animals has shown to reduce the resistance in cutting of their meat. In other species, such as sheep or poultry, Cottrell et al. [30] found a reduction in shear force in Semimembranosus muscle of Border Leicester × Merino lambs. This was observed after the administration of 500 mg/kg bolus arginine via jugular catheter, 190 min before slaughter. Furthermore, Jiao et al. [42] evaluated four dietary levels of arginine in turkey broilers (80, 100, 120, and 140% of NRC recommendation), reporting a decrease in meat shear force while increasing arginine levels. This reduction in shear force may be due to the increase in intramuscular fat since there is a negative relationship between shear force and the amount of intramuscular fat [43].

Regarding the differences of shear force between breeds, it is known that the meat produced from the Angus breed is of high quality. Angus genetics improve adipogenesis by improving marbling [44]. Additionally, its low connective tissue content (collagen) produces less shear force values, which leads to higher tenderness or reduced shear force [45]. Bureš and Bartoň [46] compared physicochemical characteristics among four different cattle breeds (Aberdeen Angus, Gascon, Holstein, and Fleckvieh), finding that Angus had the lowest shear force, better intramuscular fat content (IMF), and the highest soluble collagen content.

### 4.7. Colour

Despite the reported capacity of dietary or injected arginine to induce the transition from glycolytic muscle fibers (white) to oxidative muscle fibers (red), as mentioned in the results obtained by Chen et al. [47], the present study did not find a treatment effect on color. The results in the present study were opposite to those obtained by Tuell et al. [29], who found that a* values were higher with the inclusion of protected arginine. Moreover, they also observed that including rumen bypass arginine improved color stability, observing an increase in the a* parameter compared to the rest of the treatments. Similarly, Tous et al. [40] found an increase in redness (a*) when supplementing (Landrace × Duroc) × Pietrain pigs with 0.86% of arginine in a low protein diet. These results can be of commercial importance, due to the increase in IMF in beef without a reduction in red color, which is commonly negatively related in beef.

It is important to point out that the exposure of beef to the simulated retail display had a detrimental effect on the quality of the evaluated physicochemical characteristics of beef, as expected. This is widely documented since the presence of oxygen on the meat surface unavoidably produces first oxygenation, and later oxidation of lipids, proteins, and other components of the tissue, affecting the shelf life of the product [48]. In this regard, even some positive effects of dietary arginine in the aged beef can be reversed after seven days of exposure to oxygen.

## 5. Conclusions

The results of this work showed that the simple and low-cost microencapsulation of arginine in carnauba wax may work to introduce the amino acid into the animal system. Hence, the inclusion of microencapsulated arginine treatments in bovine diets may improve the quality of aged beef. The best results were obtained in arginine supplemented animals (both 2:1 and 3:1 ratio), by reducing the shear force and increasing the intramuscular fat content of the beef. Nevertheless, the exposure of aged beef to oxygen for 7 days can be detrimental to those positive effects. The results of this study show a potential use of a low-cost encapsulation of the amino acid for a practical and immediate use as an additive in cattle diets. Nevertheless, further research is recommended to elucidate if some factors such as different doses, longer times of supplementation, and higher experimental units can help to obtain stronger and more conclusive results, as well as improve the productive performance of the cattle. Additionally, it is recommended to further investigate the intestinal absorption of the amino acid. Supplementation with microencapsulated arginine could be a viable strategy for the livestock industry to improve meat quality.

## Figures and Tables

**Table 1:** Ingredient (%) and nutrient composition of the diet during the experiment.

Ingredient	Control	Arginine Diet
Ground dry bread	55.0	55.0
Distillery dry grain	9.6	9.6
Ground Triticale Hay	33.0	32.5
Calcium carbonate	1.2	1.2
Urea	1.0	1.0
Mineral mix	0.2	0.2
Arginine microcapsules	0.0	0.5
**Nutrient level**	**Percentage**	
DM	89.3	
Ashes	4.66	
Protein	11.69	
Total Fat	1.71	
Carbohydrates	71.21	
Kcal (100 g)	347	

**Table 2:** Productive performance of heifers from different breeds supplemented with microencapsulated arginine with carnauba wax at two different ratios.

	Treatment				Breed			
	Control	2:1	3:1	*p*	An	He	A × H	*p*
**FW**	377.8 ± 28.3	396.0 ± 46.1	395.6 ± 10.9	0.601	406.2 ± 36.0	370.0 ± 33.1	393.2 ± 9.2	0.353
**TWG**	39.6 ± 16.8	32.8 ± 18.8	26.2 ± 8.1	0.410	38.4 ± 11.9	21.8 ± 10.4	37.24 ± 18.3	0.265
**DWG**	1.88 ± 0.80	1.56 ± 0.90	1.25 ± 0.39	0.412	1.83 ± 0.57	1.04 ± 0.49	1.82 ± 0.87	0.266

FW, Final weight; TWG, Total weight gain; DWG, Daily weight gain. All variables are expressed in kg; 3:1, wax–arginine proportion; 2:1, wax–arginine proportion; An, Angus; He, Hereford; A × H, Angus × Hereford.

**Table 3:** Shear force, pH, and WHC of m. *L. lumborum* 28 d aged from heifers supplemented with microencapsulated arginine in carnauba wax at different ratios.

	Treatment				Breed			
	Control	3:1	2:1	*p*	An	He	A × H	*p*
SF	30.3 ^a^ ± 9.12	24.32 ^b^ ± 6.66	25.3 ^b^ ± 5.29	<0.001	24.02 ^b^ ± 5.78	25.59 ^b^ ± 6.57	30.4 ^a^ ± 8.92	<0.001
pH	5.32 ± 0.11	5.43 ± 0.1	5.38 ± 0.05	0.07	5.4 ± 0.06	5.38 ± 0.08	5.35 ± 0.13	0.57
WHC	62.64 ± 4.65	62.05 ± 2.75	58.29 ± 4.34	0.07	60.91 ± 6.12	61.84 ± 4.3	60.23 ± 1.76	0.71
IMF	0.72 ^b^ ± 0.44	2.12 ^a^ ± 0.99	1.16 ^b^ ± 0.71	0.002	1.59 ± 1.09	1.34 ± 0.64	1.07 ± 1.04	0.36

SF, Shear force (N); WHC, water holding capacity (%); IMF, intramuscular fat (%). 3:1, wax–arginine proportion; 2:1, wax–arginine proportion; An, Angus; He, Hereford; A × H, Angus × Hereford. ^a, b^ Different superscripts means significant differences among columns in the same line.

**Table 4:** Shear force, pH and WHC of *L. lumborum* muscle after simulated retail display exposure from heifers supplemented with microencapsulated arginine in carnauba wax at different ratios.

	Treatment				Breed			
	Control	3:1	2:1	*p*	An	He	A × H	*p*
SF	21.47 ± 4.6	20.49 ± 4.51	19.71 ± 3.33	0.12	19.12 ^b^ ± 3.82	19.71 ^b^ ± 3.43	22.94 ^a^ ± 4.41	0.0003
pH	6.37 ± 0.53	6.25 ± 0.51	6.31 ± 0.22	0.58	6.54 ^a^ ± 0.3	5.81 ^b^ ± 0.24	6.57 ^a^ ± 0.16	<0.001
WHC	69.58 ± 4.83	70.62 ± 4.18	70.19 ± 3.57	0.81	69.73 ^ab^ ± 3.56	67.53 ^a^ ± 3.99	73.14 ^b^ ± 2.76	0.01

SF. Shear force (N); WHC, water holding capacity (%); IMF, intramuscular fat (%). 3:1, wax–arginine proportion; 2:1, wax–arginine proportion; An, Angus; He, Hereford; A × H, Angus × Hereford. ^a, b^ Different superscripts means significant differences among columns in the same line.

**Table 5:** Color of m. *L. lumborum* 28 d aged from heifers supplemented with microencapsulated arginine in carnauba wax at different ratios.

	Treatments				Breed			
	Control	3:1	2:1	*p*	An	He	A × H	*p*
L*	44.24 ± 2.1	43.02 ± 1.45	44.98 ± 4.69	0.37	42.49 ± 1.03	45.47 ± 4.69	44.27 ± 1.63	0.12
a*	18.55 ± 2.91	19.66 ± 1.77	16.77 ± 1.95	0.13	16.86 ± 2.76	19.45 ± 2.23	18.67 ± 1.91	0.17
b*	11.81 ± 2.47	11.07 ± 2.39	9.88 ± 1.08	0.4	9.87 ± 1.7	11.16 ± 2.89	11.73 ± 1.35	0.41

L*, lightness; a*, redness; b*, yellowness. 3:1, wax–arginine proportion; 2:1, wax–arginine proportion; An, Angus; He, Hereford; A × H, Angus × Hereford.

**Table 6:** Color coordinates of m. *L. lumborum* after the simulated retail display exposure from heifers supplemented with microencapsulated arginine in carnauba wax at different ratios.

	Treatments				Breed			
	Control	3:1	2:1	*p*	An	He	A × H	*p*
L*	42.45 ± 1.85	43.69 ± 1	44.82 ± 1.85	0.3	43.73 ± 2.34	43.72 ± 0.84	43.51 ± 2.18	0.98
a*	16.33 ± 2.29	16.85 ± 1.56	15.81 ± 1.77	0.75	16.24 ± 2.2	16.56 ± 1.89	16.19 ± 1.06	0.95
b*	6.27 ± 0.94	7.2 ± 1.64	7.47 ± 1.58	0.11	7.06 ± 1.31	7.56 ± 1.8	6.32 ± 1.06	0.12

L*, lightness; a*, redness; b*, yellowness. 3:1, wax–arginine proportion; 2:1, wax–arginine proportion, An, Angus; He, Hereford; A × H, Angus × Hereford.

## Data Availability

The datasets generated for this study are available on request to the corresponding authors.
